# Characterization of Extra Early Spanish Clementine Varieties (*Citrus clementina* Hort ex Tan) as a Relevant Source of Bioactive Compounds with Antioxidant Activity

**DOI:** 10.3390/foods9050642

**Published:** 2020-05-16

**Authors:** Laura Cebadera, Maria Inês Dias, Lillian Barros, Virginia Fernández-Ruiz, Rosa Mª Cámara, Ángel Del Pino, Celestino Santos-Buelga, Isabel C.F.R. Ferreira, Patricia Morales, Montaña Cámara

**Affiliations:** 1 Departamento de Nutrición y Ciencia de los Alimentos, Facultad de Farmacia, Universidad Complutense de Madrid, Pza. Ramón y Cajal s/n., 28040 Madrid, Spain; lcebadera@yahoo.es (L.C.); vfernand@ucm.es (V.F.-R.); rosacama@ucm.es (R.M.C.); 2 Centro de Investigação de Montanha (CIMO), Instituto Politécnico de Bragança, Campus de Santa Apolónia, 5300-253 Bragança, Portugal; maria.ines@ipb.pt (M.I.D.); lillian@ipb.pt (L.B.); iferreira@ipb.pt (I.C.F.R.F.); 3 ANECOOP, S. Coop. C/ Monforte, 1 Entresuelo, 46010 Valencia, Spain; adelpino@anecoop.com; 4 Grupo de Investigación en Polifenoles (GIP-USAL), Facultad de Farmacia, Universidad de Salamanca, Campus Miguel de Unamuno, E-37007 Salamanca, Spain; csb@usal.es

**Keywords:** extra-early-clementine varieties, bioactive compounds, nutrients, vitamins, phenolic compounds, antioxidants

## Abstract

The most relevant nutrients and bioactive compounds (soluble sugars, dietary fiber, ascorbic acid and organic acids, individual phenolic compounds, fatty acids, and tocopherols) as well as antioxidant activity have been characterized in three extra early varieties of clementine (*Citrus clementina* Hort ex Tan. Basol, Clemensoon and Clemenrubí) cultivated in Valencia (Spain). Clementines are a relevant source of bioactive compounds, such as vitamin C (values around 80 mg/100 g), allowing to satisfy the recommended daily intake with the consumption of a normal portion. Sucrose was the most abundant sugar, and potassium the main mineral while manganese was the least. Fat content was very low (<0.5 mg/100 g), with palmitic acid and α-tocopherol the most abundant fatty acid and vitamin E form, respectively. Flavonoids were the predominant phenolic compounds, with narirutin/naringin and (neo)hesperidin the best represented ones. The antioxidant capacity evaluated by reducing power, DPPH, and β-carotene bleaching inhibition assays was satisfactory with values similar to those reported in other citrus fruits. Thus, this fruit is a relevant source of bioactive compounds with antioxidant properties of interest for consumers and the food industry.

## 1. Introduction

Several authors suggested that a diet rich in bioactive compounds with antioxidant potential is directly associated with the prevention of human diseases, like diabetes, neurological disorders, cardiovascular diseases, different types of cancer, etc. Nowadays, consumers are increasingly concerned about their health and their nutrition, increasing the intake of fruit and vegetables, which are an important source of antioxidants [1,2,3,4].

Citrus, and particularly clementines, have been consumed all over the world and are one of the most appreciated fruits due to its sweet flavor, easy peeling thin skin, and small size [5]. One drawback of the clementine is the absence of seeds, which makes them self-incompatible, having to rely on hybridization for production [6,7].

Spain is a leading producing country of small citrus fruits in Europe, being the second largest producer following China. In 2018, the production reached around 1.5 million tons, of which 70% corresponded to clementine [8].

Among the bioactive compounds present in clementines, vitamin C (ascorbic acid), citric acid, naringenin, and hesperidin stand out as the most abundant phytochemicals [9,10]. Flavanones are linked to several beneficial effects on lipid metabolism, antitumor, and antioxidant activity, among others [11]. Citric acid is known to possess important antioxidant, stabilizing, and preservative effects [5]. Regarding the lipophilic fraction, the tocopherols, which are mainly found in the peel and seeds, play an important role in the overall antioxidant activity. This antioxidant activity is due to their synergistic interaction with ascorbic acid in the lipid peroxidation inhibiting process [12].

The main purpose of the present work was to study the most relevant nutrients and bioactive compounds (soluble sugars, dietary fiber, ascorbic acid and organic acids, individual phenolic compounds, tocopherols, and fatty acids) present in three extra early varieties of clementine (*Citrus clementina* Hort ex Tan. Basol, Clemensoon and Clemenrubí) cultivated in Valencia (Spain), as an interesting source of antioxidant food ingredients.

Although studies on other non-extra early Spanish clementine varieties are available in the scientific literature [13,14]. To the authors’ best knowledge, this is the first report on the characterization of the nutritional and bioactive compounds in the current three extra early varieties.

## 2. Materials and Methods 

### 2.1. Standards and Reagents

General-use laboratory reagents (ethyl ether, CuSO_4_, HCl, HClO_4_, HNO_3_, H_2_SO_4_, K_2_SO_4_, NaOH) were analytical grade, and were supplied from Sigma–Aldrich (St. Louis, MO, USA). The eluents ethyl acetate 99.98% and n-hexane 95% of HPLC grade from Lab-Scan (Lisbon, Portugal). Methanol of analytical grade purity was purchased by Pronalab (Lisbon, Portugal). For soluble sugar characterization D(-)-fructose, D(+)-glucose, and sucrose, ≥99.5% HPLC standards were acquired from Sigma-Aldrich. For dietary fiber quantification a TDF-100A kit was acquired from Sigma-Aldrich. For fatty acids characterization the fatty acids methyl ester (FAME, standard 47885-U was obtained from Sigma-Aldrich. For mineral analysis microelements Fe(NO_3_)_3_, Cu(NO_3_)_2_, Mn(NO_3_)_2_, Zn(NO_3_)_2_ analytical standard solutions for AAS were from Panreac Química, Barcelona, Spain. For macroelements analysis NaCl, KCl, and CaCO_3_ > 99.9% purity commercial reagents, as well as Mg band, were supplied by Merck (Darmstadt, Germany), and La_2_O_3_ and CsCl (both 99% purity) were also supplied by Merck. For vitamin C and organic acids characterization ascorbic acid, oxalic, malic, citric, and fumaric acids were all from Sigma-Aldrich; and glutamic acid from Merck. For tocopherols analysis α-, β-, γ-, and δ-tocopherol ≥99.5% HPLC standards were purchased to Sigma-Aldrich and tocol was purchased from Metraya (PA, USA). The phenolic compound standards were obtained from Extrasynthese (Genay, France). For antioxidant activity 2,2-diphenyl-1-picrylhydrazyl (DPPH) was obtained from Alfa Aesar (Ward Hill, MA, USA) and trolox ((6-hydroxy-2,5,7,8-tetramethylchroman-2-carboxylic acid) was acquired from Metraya.

### 2.2. Samples Collection and Preparation

Clementine fruits from three extra early varieties of *Citrus clementina* Hort ex Tan. (Basol, Clemensoon and Clemenrubí) were gathered in their optimal ripening stage in October from a cultivar area near to Valencia (Spain) on three consecutive seasons (2011–2013). In order to obtain a representative sample, amounts of 2 kg (around 23–30 pieces) of each species were randomly selected in optimal conditions for consumption from different trees. After collection, fruits were packed and sent to the lab (UCM in Madrid, Spain) within the same day. All the samples showed a similar external appearance; the thin peel (epicarp) was removed, pulp (mesocarp) was separated and cut into small pieces, and whole fresh pulp was homogenized with an Ultra-Turrax^®^ homogenizer (IKA^®^ Works, Inc., Wilmington, NC, USA). Aliquots of homogenized fresh pulp were taken to perform physico-chemical analysis, and organic acids and vitamin C determination. One portion was immediately freeze-dried (Telstar LyoQuest freeze-dryer, Madrid, Spain) and preserved in hermetic containers at −20 °C, in a dark and dry ambient until used for the analysis of proximate composition, soluble sugars, dietary fiber, mineral composition, individual polyphenols, tocopherols, fatty acids, and antioxidant activity [15].

### 2.3. Physico-Chemical Analysis

Physico-chemical analysis in the analyzed samples were performed following AOAC procedures [16]. Dry matter (DM) was determined by desiccation, pH was measured by potentiometry (MicropH-2000, Crison Instrument, Spain), titratable acidity (TA) was quantified by titration with NaOH (0.1 N) until a pH value of 8.1, °Brix was determined according to the official methods and Ripeness index (RI) was calculated by the relation of °Brix/TA ratio (TA expressed as mg of citric acid/100 g of edible portion) [15].

### 2.4. Nutrients Characterization

#### 2.4.1. Soluble Sugars Characterization 

Soluble sugars were determined by HPLC-RI after ethanol:water (80%; *v*/*v*) extraction at 55–60 °C [17]. Equipment consists of an HPLC apparatus with an isocratic pump (PU II, Micron Analitica, SA, Spain) and a Rheodyne valve (Waters Associates, PA, USA) coupled to a RI (refractive index) detector (refractometer R401 detector, Jasco, Madrid, Spain). The chromatographic column used was a Luna 5µ NH_2_ 100 R (Phenomenex, Torrance, CA, USA). Identification and quantification were performed using Cromanec XP software (Micronec, Spain) and calibration curves (external standard method) obtained from multiple standards solutions (fructose, glucose and sucrose). Values were expressed as g/100 g fw. [15].

#### 2.4.2. Total, Soluble and Insoluble Dietary Fiber Quantification 

AOAC enzymatic–gravimetric methods (993.19 and 991.42) were used for soluble dietary fiber (SDF) and insoluble dietary fiber (IDF) analysis [16]. Total dietary fiber was calculated as the sum of insoluble and soluble dietary fiber fractions. Values were expressed as g/100 g fw of fruit edible portion.

#### 2.4.3. Total Proteins

Total nitrogen content was determined by the Kjeldahl method and further converted to protein content using a conversion factor 6.25. Values were expressed as g/100 g fw of fruit edible portion [15].

#### 2.4.4. Total Fat and Fatty Acids

Total fat was obtained after Soxhlet extraction with petroleum ether [16], and fatty acids were determined by gas-liquid chromatography with flame ionization detection (GC-FID, DANI model GC 1000 instrument, Milan, Italy) as described previously by the authors [18]. The results were processed using Clarity Software (DataApex, Prague, Czech Republic) and expressed in relative percentage (%) of each fatty acid.

#### 2.4.5. Ash Content and Mineral Composition (Macro and Microelements)

Total ash content was determined following the method 930.05 of the AOAC [16]. Briefly, 0.5 g of dried and homogenized sample was incinerated in a microwave oven with high pressure (Muffle Furnace mls1200, Monroe, LA, USA) for 24 h at 550 °C. Ashes were gravimetrically quantified, and extracted with HNO_3_ (50% *v*/*v*) and HCl (50% *v*/*v*). All measurements were performed by atomic absorption spectroscopy (Analyst 200 Perkin Elmer equipment, Waltham, USA). Microelements (Fe, Cu, Mn, and Zn) were directly measured. An additional 1/10 (*v*/*v*) dilution was performed for macroelements: LaCl_2_ (99% purity; 1.8%, *w*/*v*) for Ca and Mg determination, and CsCl_2_ (99% purity; 0.2%, *w*/*v*) for Na and K analysis. In brief, for all macroelement standards, the stock solutions (containing of 1 g/L of each element) were prepared in distilled water with a final standard solutions of 0.1–250 mg/L. La_2_O_3_ and CsCl, for Ca and Mg measurement (addition to samples and standards a solution of 58 g/L La_2_O_3_ and 87.5 g/L HCl, leading to LaCl_2_,), or for Na and K measurement (addition to samples and standards a solution of 10 g/L of CsCl). Macro and microelements were expressed as mg/100 g fw of fruit edible portion and μg/100 g fw of fruit edible portion, respectively [15].

### 2.5. Extraction and Analysis of Bioactive Compounds

#### 2.5.1. Vitamin C and Organic Acids Characterization

Both vitamin C vitamers (ascorbic and dehydroascorbic acids), as well as other individual organic acids (oxalic, tartaric, isocitric, malic, citric, fumaric, and succinic acids) were quantified by HPLC-UV after extraction with *m*-phosphoric acid (4.5%, prepared in distilled water) [5]. After *m*-phosphoric sample extraction, to an aliquot of the extracts were added L-cysteine (4 g/100 mL, from Sigma, St. Louis, USA) in order to transform the DHAA in AA for total vitamin C content quantification. DHAA was estimated by difference between total vitamin C and AA contents [15].

Separation and identification was performed by HPLC system equipped with an isocratic PU-II pump, an AS-1555 automatic injector (Jasco, Japan), a Sphereclone ODS (2) 250 × 4.60 mm, 5 µm column (Phenomenex), and a UV–VIS detector (Thermo Separation Spectra Series UV100, Madrid, Spain). The identification and quantification were performed using Cromanec XP software (Micronec, Madrid, Spain). Values were expressed as mg/100 g fw of fruit edible portion.

#### 2.5.2. Tocopherols Characterization

Tocopherols content was determined by HPLC-FL after extraction with hexane (using tocol as internal standard, IS) [19]. In brief, samples (500 mg) were homogenized with methanol and subsequently, extracted with hexane. After that, saturated NaCl aqueous solution was added, homogenized and centrifuged (Centurion K24OR refrigerated centrifuge, 6185 rpm, 5 min). The clear upper layer was transferred to a vial. The sample was re-extracted twice with hexane and the combined extracts were taken to dryness under a nitrogen stream, redissolved in n-hexane, filtered through LC filter disk, and analyzed by HPLC-FL [15].

The separation and identification were performed by HPLC (Smartline pump 1000, Knauer, Germany) connected with a FP-2020 fluorescence detector (290 nm, 330 nm; Jasco, Japan). The chromatographic separation was performed using a normal phase Polyamide II column (250, 9, 4.6 mm) from YMC Waters (Japan) at 30 °C (7971 R Grace oven). Data were analyzed using Clarity 2.4 Software (DataApex). Quantification was based on the fluorescence signal response and values were expressed as μg/100 g fw.

#### 2.5.3. Individual Phenolic Compounds Characterization

The phenolic profiles were analyzed by HPLC-DAD-ESI/MS (Hewlett-Packard 1100, Agilent Technologies, Santa Clara, USA). Samples were extraction twice with methanol:water 80:20 (*v*/*v*) at 25 °C (150 rpm, 1 h) and filtered through Whatman paper. The combined extracts were evaporated under reduced pressure at 35 °C (Büchi R-210, Flawil, Switzerland) and lyophilized (FreeZone 4.5, Labconco, KS, USA). Afterwards, each lyophilized extract was re-dissolved in 20% aqueous methanol and filtered for subsequent HPLC analysis [15,20]. 

Detection was carried out with a mass spectrometer (API 3200 Qtrap, Applied Biosystems, Darmstadt, Germany) couple to a diode array detector (DAD, 280 nm and 370 nm). Identification was performed using the chromatographic characteristics and UV-vis and mass spectra. Tentative identifications were performed by comparing the obtained information with our library and/or data reported in the literature. Quantification was achieved using calibration curves with individual standards. The results were expressed in mg/g of hydroalcoholic extract. 

### 2.6. Evaluation of the Antioxidant Capacity

The freeze-dried sample was extracted with methanol (1 h, at 25 °C) and filtered through Whatman No. 4 filter paper. The residue was then extracted with an additional portion of methanol. The combined methanolic extracts were evaporated under reduced pressure (rotary evaporator Büchi R-210, Flawil, Switzerland), re-dissolved in methanol at a concentration of 5 mg/mL, and stored at 4 °C for further use. The antioxidant capacity was evaluated using three in vitro assays: DPPH radical-scavenging activity, reducing power and β-carotene bleaching inhibition [15,21]. Results were expressed as EC_50_ values for reducing power, calculated by the interpolation from the graph of each assay. Trolox was used as positive control. 

### 2.7. Statistical Analysis

All the analyses were carried out by means of Analysis of variance (ANOVA), followed by Tukey’s test (95% confidence level). Values were expressed as means ± standard deviations (n = 3). The proximate composition, micro and macroelements and bioactive compounds were presented in three consecutive seasons and only one range of variation between seasons to simplify the interpretation of the results (n = 9). The discussion of the obtained results is based on range of variation. A multivariable analysis, canonical correlations and principal components analysis (PCA), was performed among the variables analyzed. Mean centering was used for normalization of original data. All statistical procedures were conducted using Statgraphics Centurion XVII.I software, v.17.1.12 (Warrenton, VA, USA). 

## 3. Results and Discussion

### 3.1. Nutritional and Chemical Profile of Clementine Varieties

Clementine varieties showed similar and homogeneous ranges of variation values for moisture, pH and °Brix. Variety Clemenrubí stands out by its higher acidity and ripening index value (Table 1).

**Figure 1:**
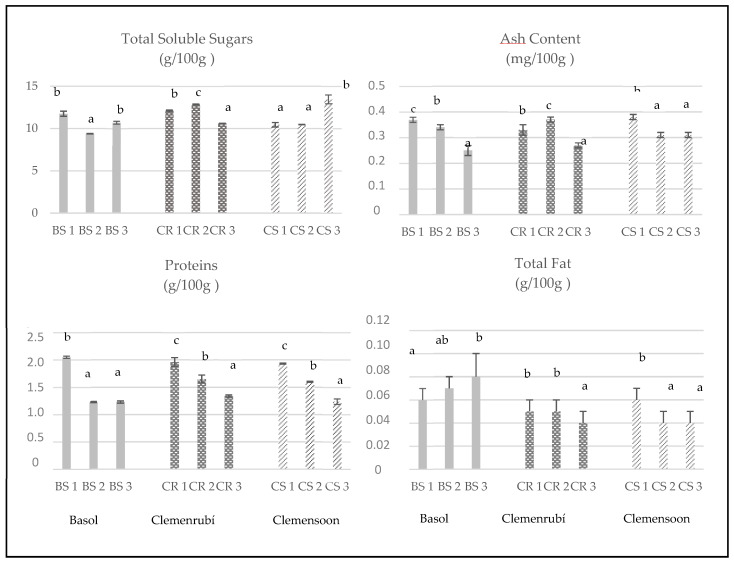
Proximate composition of extra early clementine varieties. Different letters in each column indicate statistically significant differences (*p* < 0.05) between seasons for each variety.

Results corresponding to the nutritional composition, micro, and macro elements, and hydrophilic compounds of pulp samples are shown in Table 2, Table 3, Table 4, Table 5, Table 6 and Table 7 and Figure 1. 

As expected, soluble sugars were the best-represented nutrients (9.35–13.99 g/100 g fw), followed by proteins (1.19–2.07 g/100 g fw), dietary fiber (1.04–3.66 g/100 g fw), and minerals (0.23–0.39 g/100 g fw), whereas fat was the minor compound (0.04–0.10 g/100 g fw). This leads to an energy value of these fruits in the range of 46.86–67.36 Kcal/100 g fresh fruit.

Clementine varieties showed similar values of total dietary fiber, with a different distribution between soluble (SDF) and insoluble dietary fiber (IDF) fractions, with the highest content of SDF in the Basol variety and the highest IDF content in the Clemenrubí variety (Table 2). 

According to the Food and Nutrition Board [21], the recommended dietary fiber intake should range from 19 to 31 g per day in young children (<12 years) and from 26 to 38 g per day in adolescents and adults. From our results, eating two pieces of any of the studied clementine varieties (around 170 g) would cover 8–14% of the daily recommended intake for children and 7–10% for adolescents and adults [22,23].

Related to free sugars, sucrose was the major soluble sugar, although the contents of fructose and glucose (Table 3) were also relevant and similar between them, as previously reported by [24]. 

Regarding micronutrients and starting with vitamins, vitamin C (Table 4), Clemenrubí possessed the highest amount of this vitamin with around 132 mg/100 g fw. In all cases, the predominant isoform was by far ascorbic acid (AA), which, as is well known, besides vitamin activity, also bears antioxidant properties. According to the Food and Nutrition Board, the vitamin C dietary reference intake (DRI) is 90 mg/day for adults over 19 years; from this point of view, one piece of any of the studied varieties (≈ 85 g) could cover up to 93% of DRI of this vitamin [22]. Organic acids were also analyzed in clementine varieties being citric acid, as expected, the best represented one, followed by malic, isocitric, and quinic acids with similar contents among varieties (Table 4). These results agree with the abundance of organic acids reported in other clementine varieties (tangerine *Citrus reticulata*) [25].

The mineral composition, namely microelements Cu, Fe, Mn, and Zn, and macroelements Ca, Mg, Na, and K, is shown in Table 5. Fe and Zn were the predominant microelements in clementine pulp, Basol and Clemenrubí being the varieties that presented the highest content (0.49 mg Fe/100 g fw and 0.39 mg Zn/100 g fw, respectively). As for macroelements, potassium was the most abundant one in the three varieties (up to 169.35 mg/100 g fw). These values were similar to those reported by [14] in other varieties of clementine (i.e., Clementina Fina and Clemenules). Despite the great variability of mineral content over all the years, which is highly correlated with environmental and crop conditions, we could consider these clementine varieties as interesting sources of minerals considering their higher moiety content, especially in the case of Basol and Clemenrubí varieties. Taking into account the reference intake values published by the EFSA in 2017 [26], 100 g of the edible portion of Basol clementine could cover up to 13.85% of Cu, 4.33% of Mn, and 4.26% of Mg, while a 100 g edible portion of Clemenrubí could cover up to 46.15% of Cu, 4.67% of Mn, and 4.73% of Mg. 

Despite clementine fruits presenting very low fat content (0.04–0.10 g/100 g), the authors considered it of interest to analyze the lipophilic fraction (fatty acids and tocopherols) to provide a full nutrients and bioactive compounds characterization of these non-previously studied clementine varieties. In the analyzed clementine varieties, 24 fatty acids were detected. Palmitic acid (C16:0), stearic acid (C18:0), oleic acid (C18:1n9), linoleic acid (C18:2n6), and linolenic acid (C18:3n3) were the most abundant fatty acids (Table 6). Palmitic acid was the major SFA and α-linolenic acid (ALA) was the predominant PUFA in all varieties studied, in three seasons, and oleic acid was the main monounsaturated fatty acid (MUFA). Among the fatty acid families studied, the majority families were SFA and PUFA with similar percentages, MUFA being the family with the lowest content.

Studies in other clementine varieties (i.e., variety Elarbi, from Tunisia) reported similar profile of families of fatty acids to those obtained in the present study being SFA the majority family (SFA (47.6%) < MUFA (32.54%) < PUFA (18.9%)). The main fatty acids determined in that clementine variety (Elarbi) were stearic, palmitic, oleic, linoleic and linolenic acids (representing approximately 75% of the total fatty acids), which agrees with the results obtained in the present study [27]. A similar distribution of major fatty acids was found in other citrus fruits, such as orange (*Citrus sinensis* (L.) Osbeck) [28]. 

Regarding vitamin E, α-, β-, γ-, and δ-tocopherol were detected (Table 7), with the prevalence of α-tocopherol, representing more than 90% of the vitamin E vitamers (beta, gamma, and delta tocopherol) in all the varieties studied. The results obtained from the α-tocopherol content in the analyzed clementines are comparable to those previously reported by other authors in different citrus varieties, such as sweet orange (*Citrus sinensis* (L.) Osbeck) [29]. 

### 3.2. Individual Phenolic Composition and Antioxidant Capacity of Clementine Fruits

Fourteen phenolic compounds were detected and tentatively identified in the studied samples, together with three other non-phenolic phytochemicals: dihydrophaseic acid and its glucoside and nomilin-glucoside. The peak characteristics (retention time, λmax in the visible region, and mass spectral data) are shown in Supplementary Materials Table S1. Tentative identifications and quantification of phenolic compounds in the hydromethanolic extracts of the three clementine varieties are shown in Supplementary Materials Table S2.

Flavonoids (peaks 5, 6, 9, 13–15, and 17) represented the largest family of phenolic compounds in the three varieties, being most of them identified as *C*- and *O*-glycosides, as expected in citrus varieties [30,31,32]. Peak 5 presented a pseudomolecular ion [M-H]^−^ at *m/z* 593, releasing [33]. MS^2^ fragments at *m/z* 503 (−90 u), 473 (−120 u), 383 (−120–90 u), and 353 (−120–120 u), a fragmentation pattern typical of *C*-glycosyl-flavones [33]. A compound with similar characteristics was previously identified as vicenin II (apigenin-6,8-di-*C*-glucoside), as the major phenolic constituent present in the citrus fruit juices [31]. Peak 9 presented a pseudomolecular ion [M-H]^−^ at *m/z* 623 and was tentatively identified as diosmetin-6,8-di-*C*-glucoside. The presence of this latter *C*-glycosyl flavone has also been reported in *Citrus suhuiensis* and *Citrus microcarpa* fruits and citrus juices [31,34]. The other detected flavonoids would correspond to *O*-linked glycosides. Peak 13 was identified as rutin (quercetin-3-*O*-rutinoside) by comparison of its UV, mass spectrum, and retention time with a commercial standard. Mass spectral characteristics of peak 14, with [M-H]^−^ at *m/z* 579 releasing a unique MS^2^ fragment at *m/z* 271 (−308 u, loss of a deoxyhexosyl-hexoside moiety) would match both naringenin-7-*O*-rutinoside (narirutin) and naringenin-7-*O*-neohesperidoside (naringin), which are common flavanones in citrus fruits [1,32,35,36,37]. Similarly, peak 15, with [M-H]^−^ at *m/z* 609 releasing an MS^2^ fragment at *m/z* 301, that could either correspond to hesperetin-7-*O*-rutinoside (hesperidin) or hesperetin-7-*O*-neohesperidoside (neohesperidin). Data obtained from HPLC-DAD-ESI/MS analysis do not allow to identify the precise identity of those compounds. Peak 17 ([M-H]^−^ at *m/z* 593) released an MS^2^ fragment at *m/z* 285 [kaempferol-H]^−^ also from the loss of −308 u (deoxyhexosyl-hexoside), although in this case, it was tentatively assigned to a kaempferol-*O*-deoxyhexosyl-hexoside. As for peak 6 ([M-H]^−^ at *m/z* 433, fragment ion at *m/z* 271) a tentative identification as naringenin-*O*-hexoside was proposed. 

Concerning phenolic acids derivatives (peaks 1–4 and 10–12), peak 1 was tentatively identified as sinapoyl-glucoside, based on its pseudomolecular ion ([M-H]^−^ at *m/z* 385) and previous identification in *C. limetta* peel [32]. Peak 2 presented the same pseudomolecular ion as peak 1 but with a different fragmentation pattern. A compound with similar spectral characteristics was reported by [38] in the medicinal plant *Mercurialis perennis* L. and identified as feruloyl glucarate; the same compound (*p*-feruloyl-glucaric acid) and the analogous *p*-feruloyl-galactaric acid were also isolated from orange peel and fully identified by [39], thus, the same assumption was made for the herein-studied fruits. Mass spectral characteristics of peak 4 ([M-H]^−^ at *m/z* 355 and MS^2^ fragments at *m/z* 295, 235 and 193) were in accordance with those described by [38] for feruloyl-6′-*O*-glucose found in *C. limetta* peel and previously identified by [40] in lemon juice, so that this identity was also assumed for the compound detected herein. Peak 3, with [M-H]^−^ at *m/z* 341 presenting an MS^2^ fragment at *m/z* 179 (caffeic acid, −162 u), could correspond to a caffeoyl-hexoside. Peaks 11 and 12, with the same pseudomolecular ion [M-H]^−^ at *m/z* 561 and an MS^2^ fragmentation pattern (*m/z* at 367, 191, 193, 173, and 134) coherent with feruloylquinic acid (368 Da); the mass difference (194 u) might correspond to dihydroferulic acid (196 Da) taking into account a loss of two mass units in the intermolecular linkage. Overall, these compounds were tentatively assigned as dihydroferulic-feruloylquinic acid dimers. Peak 10 ([M-H]^−^ at *m/z* 427) should also correspond to a hydroxycinnamoyl derivative as suggested by its UV spectrum and the observation of characteristic MS^2^ fragments at *m/z* 385, 367, 223, and 179, although a precise identity could not be established.

The UV and mass characteristics of peak 16 ([M-H]^−^ 477 at *m/z* 693) coincide with those of nomilin glucoside, a non-phenolic compound belonging to the family limonoids, largely reported in citrus fruits [32,41]. As for peak 7 ([M-H]^−^ at *m/z* 443), mass concordance has been found with the compound dihydrophaseic acid glucoside [42], a metabolite from the abscisic acid pathway identified in the peel of *Citrus kawachiensis* by [36]. A compound with the same mass characteristics was also found by [32] in the peel of *C. limetta*, although those authors did not propose a tentatively identification for the compound. Finally, peak 8 ([M-H]^−^ at *m/z* 281) could correspond to the aglycone of peak 7, dehydrophaseic acid. The three extra early clementine varieties revealed high phenolic contents, being flavonoids the most abundant group (Table 8) with the flavanones narirutin/naringin and (neo) hesperidin as the main individual compounds.

Table 9 displays the antioxidant activity of the clementine extracts. In general terms, Basol variety showed the highest antioxidant activity measured by the reducing power assay against oxidative stress (EC_50_ values 1.32–2.46 mg/mL methanolic extract) and the β-carotene bleaching inhibition (0.21–0.82 mg/mL), while Clemensoon presented greater antioxidant activity measured through the DPPH assay (3.36–10.98 mg/mL methanolic extract), suggesting greater ability to avoid the formation of and/or scavenging oxidizing substances in the hydrophilic phase. 

The interpretation of the data of antioxidant activity of foods is complex, due to the variety of assays available. As different antioxidant activity assays measure their activity through mechanisms, the combined analysis of samples through different assays is often needed to have an overall idea of the antioxidant properties of the samples [43]. In order to assess which bioactive compounds present in the analysed clementine varieties are responsible for their antioxidant activity, a correlation analysis has been carried out. Related to antioxidant activity assays, DPPH assay provide useful information about clementines scavenging activity. Positive correlations (*p* < 0.05) were observed between EC_50_ values from DPPH method results and vitamin C (0.4032; 0.0370); phenolic compounds (0.6647; 0.0002), which are mainly compound by sinapoyl-glucoside and caffeoyl-hexoside; flavonoids (0.7188; 0.0002), which are mainly compound by narirutin/naringin and hesperidin/neohesperidin, and PUFA (0.5023; 0.0076) mainly compound by linoleic and α-linolenic acid.

Similarly, β-carotene bleaching inhibition assay was highly and positively correlated with vitamin C (0.5132; 0.0062), flavonoids (0.5279; 0.0047) and phenolic acids (0.4943; 0.0088), which is indicative that these compounds are also active against the lipid peroxidation process. Furthermore, vitamin C is strongly correlated with α-tocopherol (0.4120; 0.0327), which would be indicative of the protective effect that vitamin C (ascorbic acid) exerts against the oxidation of free radicals. Other relevant correlations were observed by α-tocopherol and PUFA (−0.6646; 0.0002), thus avoiding its lipid peroxidation measured by β-carotene bleaching inhibition assay. This tendency was also reported by [43].

As it can be seen in Table 9, a great variability of EC_50_ values was found between seasons, this could be explained due to the great variability also observed in their bioactive compounds content. Therefore, we can affirm that these bioactive compounds are the mains responsible for the antioxidant activities of clementine varieties, being useful in the prevention of several pathologies as previously reported by [44].

Overall, the antioxidant activity of all the samples can be considered satisfactory and similar with results reported in other citrus fruits like sweet orange [29,45] or pummel (*Citrus grandis* Osbeck) [1,46].

### 3.3. PCA Analysis of Clementine Fruits

In order to reduce the multidimensional structure of the data, a principal component analysis (PCA) was performed, which provided a two-dimensional map for explaining the observed variance. All the studied samples were plotted on the reduced space of the two principal components. The two components of the PCA performed explain 99.85% of the total variance (55.33% first and 44.51% second). The first principal component is highly and positively correlated to α-tocopherol (0.312771), soluble fiber (0.2957), and DPPH assay (0.2960), and negatively and highly correlated with PUFA (−0.3010), insoluble fiber (−0.2963) and inhibition of discoloration of β-carotene assay (−0.3068). The second principal component was strongly and positively correlated to soluble sugars, mainly sucrose (0.3523) and reducing power assay (0.3018), and negatively with citric acid (−0.3161), phenolic compounds, flavonoids (−0.3359), phenolic acids (−0.3338), and Fe (−0.3068).

**Figure 2:**
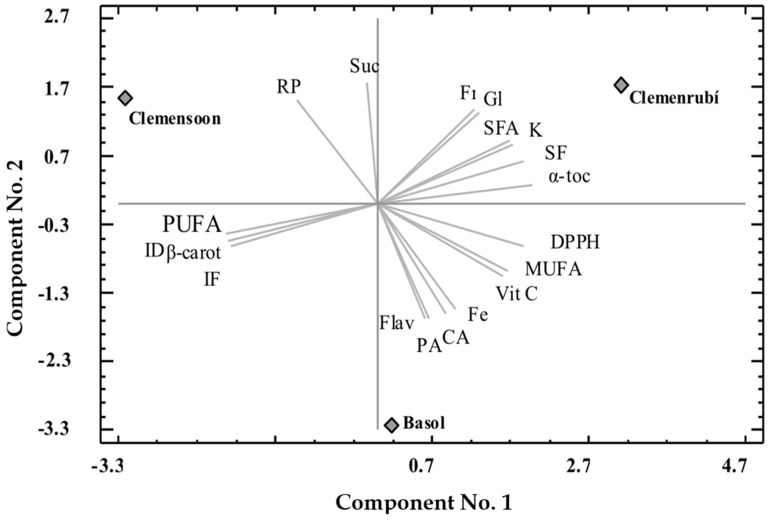
Principal component analysis (PCA) projection of two principal components. Vit C: vitamin C; α-toc: α-tocopherol; PA: phenolic acids; Flav: total flavonoids; CA: citric acid; SFA: saturated fatty acids; MUFA: monounsaturated fatty acids; PUFA: polyunsaturated fatty acids; Fr: fructose; Gl: glucose; Suc: sucrose; Fe: iron; K: potassium; IF: insoluble fiber; SF: soluble fiber; DPPH: DPPH assay; PR: reduction power assay; ID β-carot: inhibition of discoloration of β-carotene.

All the studied clementine varieties are plotted on the reduced space of the two first principal components as shown in Figure 2. As it can be seen, the Basol variety was negatively characterized by the second principal component. Thus, this variety is highlighted by higher phenolic compounds, both flavonoids and phenolic acids, Fe and citric acid content, as well as by lower soluble sugars), contrarily to Clemenrubí, which was positively correlated with the first principal component. Clemensoon was negatively characterized by the first principal component and, therefore, is characterized by higher insoluble fiber and polyunsaturated fatty acids content and higher antioxidant activity measured by the inhibition method of discoloration of β-carotene. To the contrary, this variety is characterized by a lower α-tocopherol and soluble fiber content. 

## 4. Conclusions

This work reports for the first time the phytochemical composition and antioxidant activity of some extra early clementine varieties (*Citrus clementina* Hort ex Tan. cv. Basol, Clemensoon and Clemenrubí) natives from Spain. It is important to highlight the presence of relevant levels of vitamin C (mainly ascorbic acid) that allow meeting the dietary reference intake (DRI, 80 mg/100 g) of this vitamin through the consumption of a normal portion of any of the studied clementines (around 75 g). These varieties also contained relatively high levels of total dietary fiber, with outstanding levels of the insoluble fraction in Clemenrubí variety. Furthermore, they also presented significant phenolic contents, being flavonoids the most abundant group of phenolic compounds, particularly the flavanones narirutin/naringin and (neo) hesperidin. These clementine varieties are revealed as good sources of bioactive compounds with potential promoting health benefits that could be of great interest for the food industry in order to develop foods enriched in natural compounds as well as to improve food antioxidant capacity.

## Figures and Tables

**Table 1:** Physicochemical characteristics (range of variation) of extra early clementine varieties.

Clementine Varieties	Moisture g/100 g	pH	TA	°Brix	RI
Basol	84.37–88.05	3.02–3.19	11.51–15.84	11.90–14.80	13.91–17.91
Clemenrubí	82.46–86.46	3.02–3.32	11.26–19-83	12.69–14.89	10.06–20.48
Clemensoon	85.56–86.52	3.01–3.43	12.27–13.18	12.02–13.20	14.52–16.47

TA: titratable acidity; RI: ripeness index.

**Table 2:** Dietary fiber composition (g/100 g fw of the fruit edible portion) of extra early clementine varieties.

Clementine Varieties	Season	Insoluble Fiber	Soluble Fiber	Total Fiber
Basol	1	0.47 ± 0.04 ^a^	0.57 ± 0.10 ^a^	1.07 ± 0.03 ^a^
	2	1.00 ± 0.05 ^b^	1.15 ± 0.03 ^c^	2.13 ± 0.05 ^b^
	3	2.14 ± 0.11 ^c^	1.01 ± 0.09 ^b^	3.45 ± 0.21 ^c^
	RV	0.43–2.25	**0.47–1.18**	**1.04–3.66**
Clemenrubí	1	0.74 ± 0.06 ^a^	0.69 ± 0.01 ^a^	1.46 ± 0.03 ^a^
	2	1.20 ± 0.10 ^c^	1.08 ± 0.05 ^b^	2.36 ± 0.22 ^b^
	3	0.96 ± 0.12 ^b^	1.75 ± 0.14 ^c^	2.98 ± 0.26 ^c^
	RV	0.68–1.30	**0.68–1.89**	**1.43–3.24**
Clemensoon	1	1.14 ± 0.10 ^a^	0.55 ± 0.04 ^a^	1.70 ± 0.03 ^a^
	2	1.11 ± 0.10 ^a^	1.07 ± 0.09 ^b^	2.18 ± 0.10 ^b^
	3	1.66 ± 0.15 ^b^	1.06 ± 0.11 ^b^	2.71 ± 0.24 ^c^
	RV	1.01–1.81	0.51–1.17	1.67–2.95

Total fiber result from the sum of soluble and insoluble fractions. RV: Range of Variation. Mean ± standard deviation (*n* = 9). In each column, the different letters (a,b,c) indicate statistically significant differences (*p* < 0.05) between seasons for each variety.

**Table 3:** Soluble sugars (mg/100 g fw of fruit edible portion) in extra early clementine varieties.

Clementine Varieties	Season	Soluble Sugars		
		Fructose	Glucose	Sucrose
Basol	1	2.86 ± 0.08 ^b^	2.65 ± 0.07 ^b^	6.24 ± 0.15 ^c^
	2	2.36 ± 0.04 ^a^	2.27 ± 0.02 ^a^	4.75 ± 0.07 ^a^
	3	2.88 ± 0.05 ^b^	2.90 ± 0.04 ^c^	4.93 ± 0.07 ^b^
	RV	2.32–2.94	2.25–2.94	4.68–6.39
Clemenrubí	1	3.07 ± 0.02 ^b^	3.03 ± 0.01 ^b^	6.01 ± 0.04 ^b^
	2	3.19 ± 0.06 ^c^	3.22 ± 0.05 ^c^	6.45 ± 0.11 ^c^
	3	2.64 ± 0.01 ^a^	2.63 ± 0.02 ^a^	5.34 ± 0.02 ^a^
	RV	2.63–3.25	2.61–3.27	5.32–6.56
Clemensoon	1	2.39 ± 0.05 ^a^	2.27 ± 0.07 ^a^	5.78 ± 0.14 ^a^
	2	2.40 ± 0.01 ^a^	2.37 ± 0.02 ^b^	5.70 ± 0.01 ^a^
	3	3.59 ± 0.13 ^b^	3.50 ± 0.13 ^c^	6.38 ± 0.24 ^b^
	RV	2.34–3.72	2.20–3.63	4.64–6.62

RV: Range of Variation. Mean ± standard deviation (*n* = 9). In each column, the different letters (a,b,c) indicate statistically significant differences (*p* < 0.05) between seasons for each variety.

**Table 4:** Vitamin C (total, ascorbic and dehidroascorbic acid) and other organic acids (mg/100 g fw of fruit edible portion) in extra early clementine varieties.

Clementine Variety	Compound	Season 1	Season 2	Season 3	RV
Basol	Ascorbic acid	103.08 ± 3.40 ^b^	91.48 ± 3.43 ^a^	116.24 ± 3.24 ^c^	88.05–119.48
	Dehidroascorbic acid	2.30 ± 0.36 ^a^	9.86 ± 0.22 ^c^	5.85 ± 0.65 ^b^	1.94–10.08
	Total vitamin C	106.69 ± 3.96 ^a^	101.01 ± 2.95 ^a^	118.30 ± 8.95 ^a^	98.06–127.25
	Citric acid	515.72 ± 6.98 ^b^	482.81 ± 17.24 ^a^	664.79 ± 5.39 ^c^	465.57–680.18
	Fumaric acid	0.50 ± 0.01 ^b^	0.79 ± 0.04 ^c^	0.04 ± 0.00 ^a^	0.04–0.83
	Isocitric acid	92.20 ± 3.12 ^b^	150.33 ± 5.06 ^c^	53.79 ± 2.74 ^a^	51.05–155.39
	Malic acid	113.24 ± 1.21 ^c^	119.41 ± 3.47 ^b^	79.92 ± 6.86 ^a^	73.03–122.88
	Oxalic acid	52.48 ± 2.20 ^c^	41.04 ± 2.25 ^b^	28.71 ± 1.70 ^a^	27.01–54.68
	Quinic acid	92.52 ± 3.68 ^c^	45.19 ± 3.49 ^a^	71.69 ± 1.88 ^b^	41.70–96.20
Clemenrubí	Ascorbic acid	120.04 ± 3.32 ^b^	91.26 ± 2.95 ^a^	94.61 ± 5.81 ^a^	88.31–123.36
	Dehidroascorbic acid	7.04 ± 0.06 ^b^	5.36 ± 0.94 ^a^	5.04 ± 0.01 ^a^	4.42–7.12
	Total vitamin C	127.12 ± 4.92 ^b^	94.70 ± 1.37 ^a^	100.05 ± 8.15 ^a^	93.33–132.04
	Citric acid	582.63 ± 41.86 ^b^	561.01 ± 8.80 ^b^	446.39 ± 3.20 ^a^	449.59–623.86
	Fumaric acid	0.60 ± 0.03 ^b^	0.77 ± 0.03 ^c^	0.04 ± 0.01 ^a^	0.03–0.80
	Isocitric acid	63.21 ± 0.10 ^b^	73.34 ± 3.76 ^c^	49.56 ± 5.18 ^a^	44.38–77.10
	Malic acid	93.76 ± 5.01 ^a^	137.12 ± 4.30 ^c^	115.49 ± 8.09 ^b^	88.75–141.42
	Oxalic acid	64.03 ± 3.35 ^c^	43.10 ± 1.80 ^b^	30.12 ± 1.36 ^a^	28.76–67.38
	Quinic acid	90.29 ± 3.37 ^c^	40.89 ± 3.07 ^a^	71.65 ± 6.24 ^b^	37.82–93.66
Clemensoon	Ascorbic acid	90.86 ± 11.45 ^a^	96.20 ± 4.18 ^a^	100.60 ± 9.52 ^a^	79.41–110.12
	Dehidroascorbic acid	10.19 ± 0.18 ^b^	4.55 ± 0.83 ^a^	4.61 ± 0.80 ^a^	3.72–10.37
	Total vitamin C	99.28 ± 5.91 ^a^	93.79 ± 4.83 ^a^	105.21 ± 0.79 ^a^	88.96–106.00
	Citric acid	349.83 ± 16.44 ^a^	548.34 ± 23.84 ^b^	639.37 ± 35.89 ^c^	333.39–675.26
	Fumaric acid	0.67 ± 0.04 ^b^	0.99 ± 0.07 ^c^	0.04 ± 0.00 ^a^	0.04–1.06
	Isocitric acid	99.83 ± 5.73 ^c^	69.10 ± 3.20 ^b^	47.54 ± 2.76 ^a^	44.78–105.56
	Malic acid	121.40 ± 9.72 ^b^	126.36 ± 3.63 ^b^	99.34 ± 1.95 ^a^	97.39–129.99
	Oxalic acid	50.13 ± 4.64 ^c^	40.84 ± 2.13 ^b^	30.70 ± 0.61 ^a^	30.09–54.77
	Quinic acid	73.53 ± 4.81 ^b^	38.82 ± 2.23 ^a^	73.44 ± 6.71 ^b^	36.59–78.34

RV: Range of Variation. Mean ± standard deviation (*n* = 9). In each column, the different letters (a,b,c) indicate statistically significant differences (*p* < 0.05) between seasons for each variety.

**Table 5:** Micro and macro elements (mg/100 g fw of fruit edible portion) in extra early clementine varieties (mean ± SD, *n* = 9).

		Microelements				Macroelements			
Clementines Varieties	Season	Cu	Fe	Mn	Zn	Ca	Mg	K	Na
Basol	1	0.05 ± 0.01 ^a^	0.45 ± 0.01 ^b^	0.12 ± 0.01 ^c^	0.12 ± 0.01 ^a^	20.01 ± 1.57 ^b^	12.03 ± 0.74 ^b^	140.98 ± 1.72 ^c^	4.79 ± 0.57 ^a^
	2	0.18 ± 0.01 ^c^	0.23 ± 0.01 ^a^	0.02 ± 0.01 ^b^	0.26 ± 0.02 ^c^	24.05 ± 2.13 ^b^	11.27 ± 1.07 ^b^	99.96 ± 0.72 ^b^	7.10 ± 0.02 ^b^
	3	0.07 ± 0.01 ^b^	0.46 ± 0.03 ^b^	0.01 ± 0.01 ^a^	0.15 ± 0.01 ^b^	11.64 ± 1.13 ^a^	6.96 ± 0.13 ^a^	68.48 ± 0.53 ^a^	16.81 ± 0.43 ^c^
	RV	0.05–0.18	0.22–0.49	0.01–0.13	0.12–0.28	10.51–26.18	6.83–12.77	67.95–142.70	4.22–17.24
Clemenrubí	1	0.04 ± 0.01 ^a^	0.30 ± 0.05 ^b^	0.14 ± 0.01 ^c^	0.15 ± 0.01 ^a^	23.16 ± 0.77 ^b^	13.01 ± 1.18 ^c^	161.09 ± 8.26 ^c^	8.55 ± 0.62 ^b^
	2	0.14 ± 0.02 ^c^	0.36 ± 0.01 ^b^	0.05 ± 0.01 ^b^	0.36 ± 0.03 ^b^	22.36 ± 1.24 ^b^	9.67 ± 0.66 ^b^	106.04 ± 3.92 ^b^	5.11 ± 0.43 ^a^
	3	0.07 ± 0.01 ^b^	0.24 ± 0.02 ^a^	0.01 ± 0.01 ^a^	0.15 ± 0.01 ^a^	14.47 ± 1.58 ^a^	7.40 ± 0.62 ^a^	63.59 ± 1.45 ^a^	10.45 ± 0.44 ^c^
	RV	0.04–0.6	0.22–0.37	0.01–0.14	0.14–0.39	12.89–23.93	6.78–14.19	62.14–169.35	4.68–10.95
Clemensoon	1	0.05 ± 0.01 ^b^	0.20 ± 0.02 ^a^	0.09 ± 0.01 ^b^	0.07 ± 0.01 ^a^	15.42 ± 0.50 ^a^	9.45 ± 0.43 ^a^	122.52 ± 3.95 ^c^	8.22 ± 0.86 ^a^
	2	0.02 ± 0.01 ^a^	0.21 ± 0.01 ^a^	nd	0.13 ± 0.06 ^b^	21.94 ± 1.86 ^b^	9.61 ± 0.72 ^a^	93.59 ± 1.50 ^b^	7.76 ± 0.01 ^b^
	3	0.06 ± 0.01 ^b^	0.30 ± 0.02 ^b^	0.01 ± 0.01 ^a^	0.11 ± 0.01 ^b^	21.45 ± 1.94 ^b^	9.17 ± 0.52 ^a^	68.90 ± 4.04 ^a^	9.01 ± 0.79 ^c^
	RV	0.02–0.07	0.18–0.32	nd–0.10	0.06–0.19	14.92–23.80	8.65–10.33	64.86–126.47	7.75–9.80

RV: Range of variation; nd: Non- detected; tr: traces (<0.01 mg/100 g fw). Mean ± Standard deviation (*n* = 9). In each column, the different letters (a,b,c) indicate statistically significant differences (*p* < 0.05) between seasons for each variety.

**Table 6:** Fatty acids (relative %), total fat (g/100 g fw of fruit edible portion) in extra early clementine varieties (mean ± SD, *n* = 9).

**Main FA**	**Basol**	**Clemenrubí**	**Clemensoon**
	**Season 1**		
C16:0	24.29 ± 0.43 ^c^	34.50 ± 0.34 ^a^	26.38 ± 0.14 ^b^
C18:0	5.73 ± 0.21 ^b^	8.31 ± 0.00 ^a^	5.92 ± 0.61 ^b^
C18:1n9	8.42 ± 0.18 ^a^	8.09 ± 0.03 ^a^	6.85 ± 0.49 ^b^
C18:2n6	18.86 ± 0.19 ^a^	16.91 ± 0.18 ^b^	19.30 ± 0.09 ^a^
C18:3n3	27.32 ± 0.13 ^b^	20.43 ± 0.06 ^c^	28.29 ± 0.34 ^a^
Total Fat (g/100 g fw)	0.05 ± 0.00 ^b^	0.06 ± 0.00 ^a^	0.06 ± 0.00 ^a^
SFA (%)	40.98 ± 0.41 ^c^	52.21 ± 0.19 ^a^	42.57 ± 1.22 ^b^
MUFA (%)	11.18 ± 0.26 ^a^	10.08 ± 0.07 ^b^	9.29 ± 0.44 ^c^
PUFA (%)	47.84 ± 0.15 ^a^	37.72 ± 0.25 ^b^	48.13 ± 1.66 ^a^
**Main FA**	**Season 2**		
C16:0	27.21 ± 0.86 ^a^	26.34 ± 1.99 ^a^	28.38 ± 0.33 ^a^
C18:0	4.20 ± 0.44 ^b^	6.18 ± 0.06 ^a^	6.50 ± 0.03 ^a^
C18:1n9	9.45 ± 0.83 ^b^	9.84 ± 1.59 ^b^	13.72 ± 0.44 ^a^
C18:2n6	14.26 ± 0.17 ^a^	15.62 ± 1.03 ^a^	15.42 ± 0.10 ^a^
C18:3n3	30.88 ± 1.55 ^a^	23.72 ± 1.26 ^b^	18.39 ± 0.25 ^c^
Total Fat (g/100 g fw)	0.06 ± 0.01 ^a^	0.05 ± 0.00 ^a^	0.04 ± 0.00 ^b^
SFA (%)	41.00 ± 0.63 ^b^	46.59 ± 2.66 ^a^	47.37 ± 0.08 ^a^
MUFA (%)	13.32 ± 1.13 ^b^	12.92 ± 1.26 ^b^	15.38 ± 0.09 ^a^
PUFA (%)	45.69 ± 1.76 ^a^	40.49 ± 1.99 ^b^	37.25 ± 0.17 ^b^
**Main FA**	**Season 3**		
C16:0	26.48 ± 0.72 ^a^	27.72 ± 0.15 ^a^	25.03 ± 1.12 ^a^
C18:0	9.70 ± 0.62 ^a^	9.18 ± 0.55 ^a^	7.92 ± 1.27 ^b^
C18:1n9	15.64 ± 2.11 ^a^	12.87 ± 0.36 ^b^	9.67 ± 2.13 ^c^
C18:2n6	13.59 ± 2.69 ^a^	6.95 ± 0.69 ^b^	8.81 ± 2.54 ^b^
C18:3n3	9.21 ± 0.33 ^c^	13.80 ± 1.14 ^b^	24.49 ± 8.85 ^a^
Total Fat (g/100 g fw)	0.08 ± 0.02 ^a^	0.04 ± 0.00 ^b^	0.04 ± 0.00 ^b^
SFA (%)	59.90 ± 4.33 ^a^	62.96 ± 1.33 ^a^	53.98 ± 9.7 ^a^
MUFA (%)	16.78 ± 2.05 ^a^	15.21 ± 0.30 ^a^	11.87 ± 1.69 ^b^
PUFA (%)	23.33 ± 2.29 ^a^	21.83 ± 1.63 ^a^	34.15 ± 11.48 ^a^

FA: Fatty acids; SFA: saturated fatty acids; MUFA: monounsaturated fatty acids; PUFA: polyunsaturated fatty acids. In each row, the different letters (a,b,c) indicate statistically significant differences (*p* < 0.05) between seasons for each variety.

**Table 7:** Tocopherols content (μg/100 g fw of fruit edible portion) in extra early clementine varieties (mean ± SD, *n* = 9).

Tocopherol Vitamers	Basol	Clemenrubí	Clemensoon
**Season 1**			
α-tocopherol	201.87 ± 26.72 ^a^	219.27 ± 4.60 ^a^	141.55 ± 2.83 ^b^
β-tocopherol	6.23 ± 0.43 ^a^	5.86 ± 0.01 ^a^	3.24 ± 0.22 ^b^
γ-tocopherol	3.33 ± 0.97 ^a^	3.23 ± 0.00 ^a^	4.06 ± 0.09 ^a^
δ-tocopherol	nd	nd	nd
Total Tocopherols	211.43 ± 26.18 ^a^	228.36 ± 4.61 ^a^	148.85 ± 2.96 ^b^
**Season 2**			
α-tocopherol	103.14 ± 2.10 ^b^	121.90 ± 5.14 ^b^	135.01 ± 2.31 ^a^
β-tocopherol	5.99 ± 0.20 ^b^	9.10 ± 0.00 ^a^	8.20 ± 0.00 ^a^
γ-tocopherol	nd	4.26 ± 0.01	2.97 ± 0.01
δ-tocopherol	2.16 ± 0.10	nd	nd
Total Tocopherols	111.29 ± 2.41 ^b^	135.26 ± 5.15 ^a^	146.18 ± 2.32 ^a^
**Season 3**			
α-tocopherol	232.08 ± 0.00 ^b^	302.37 ± 3.71 ^a^	192.26 ± 8.32 ^c^
β-tocopherol	nd	nd	nd
γ-tocopherol	7.14 ± 0.10 ^b^	17.41 ± 0.32 ^a^	8.97 ± 0.00 ^b^
δ-tocopherol	11.21 ± 0.10 ^a^	8.32 ± 0.21 ^b^	6.31 ± 0.10 ^c^
Total Tocopherols	250.43 ± 0.1 ^b^	328.10 ± 4.24 ^a^	207.54 ± 8.33 ^b^

nd—non detected. In each row different letters (a,b,c) mean significant differences (*p* < 0.05).

**Table 8:** Quantification (mg/g of methanolic extract) of the main phenolic compounds in extra early clementine varieties (mean ± SD, *n* = 9).

Season	Clementine Varieties	Tentative Identification					Total Flavonoids	Total Phenolic Acids	Total Phenolic Compounds
		Sinapoyl-glucoside ^1^	Caffeoyl-hexoside ^3^	Vicenin II (apigenin-6,8-di-C-glucoside) ^4^	Narirutin/Naringin ^5^	Hesperidin/Neohesperidin ^9^			
1	BS	3.4 ± 0.20	1.56 ± 0.03	2.80 ± 0.01	4.15 ± 0.01	4.52 ± 0.02	13.81 ± 0.02 ^b^	10.51 ± 0.50 ^b^	25.27 ± 0.53 ^b^
	CR	2.04 ± 0.01	1.58 ± 0.07	0.88 ± 0.01	2.563 ± 0.011	2.98 ± 0.02	7.33 ± 0.04 ^c^	7.45 ± 0.21 ^c^	15.35 ± 0.21 ^c^
	CS	3.92 ± 0.01	3.26 ± 0.07	1.84 ± 0.01	5.82 ± 0.01	5.25 ± 0.02	15.46 ± 0.01 ^a^	15.73 ± 0.07 ^a^	32.78 ± 0.08 ^a^
2	BS	1.09 ± 0.01	1.03 ± 0.02	0.42 ± 0.01	0.80 ± 0.0	0.51 ± 0.01	3.36 ± 0.02 ^b^	2.58 ± 0.02 ^b^	5.94 ± 0.01 ^b^
	CR	1.40 ± 0.04	0.90 ± 0.01	0.46 ± 0.01	0.91 ± 0.01	0.65 ± 0.01	3.53 ± 0.01 ^a^	2.84 ± 0.02 ^a^	6.37 ± 0.01 ^a^
	CS	0.88 ± 0.01	0.72 ± 0.01	0.37 ± 0.01	0.75 ± 0.01	0.34 ± 0.01	2.66 ± 0.01 ^c^	2.24 ± 0.01 ^c^	4.91 ± 0.01 ^c^
3	BS	1.20 ± 0.02	1.09 ± 0.02	0.84 ± 0.01	0.81 ± 0.01	0.59 ± 0.01	3.67 ± 0.01 ^a^	3.10 ± 0.01 ^a^	6.78 ± 0.01 ^a^
	CR	0.93 ± 0.01	0.93 ± 0.03	0.69 ± 0.02	0.76 ± 0.01	0.54 ± 0.01	3.17 ± 0.04 ^b^	2.86 ± 0.05 ^b^	6.03 ± 0.09 ^b^
	CS	0.90 ± 0.01	0.85 ± 0.01	0.63 ± 0.01	0.63 ± 0.01	0.39 ± 0.01	2.66 ± 0.01 ^c^	2.28 ± 0.02 ^c^	4.94 ± 0.03 ^c^

BS (Basol), CR (Clemenrubí), CS (Clemensoon). nq—not quantified. Calibration curves used: 1: sinapic acid (y = 270.42x + 62.29; *R*^2^ = 0.999); 3: caffeic acid (y = 359.01x + 488.40; *R*^2^ = 0.998); 4: apigenin-6-*C*-glucoside (y = 179.52x + 116.83; *R*^2^ = 0.999); 5: naringenin (y = 539.98x + 161.46; *R*^2^ = 0.995); 9: hesperetin (y = 792.22x − 76.88; *R*^2^ = 0.999). In each row per season different letters (a,b,c) mean significant differences (*p* < 0.05).

**Table 9:** Antioxidant activity of the methanolic extracts (EC_50_, mg/mL methanolic extract) of the three studied extra early clementine varieties (mean ± SD, *n* = 9).

Clementine Variety	Season	DPPH Assay	Reducing Power Assay	β-Carotene Bleaching Inhibition
Basol	1	6.06 ± 0.01 ^b^	1.52 ± 0.01 ^b^	0.21 ± 0.01 ^a^
	2	5.27 ± 0.37 ^a^	1.32 ± 0.01 ^a^	0.30 ± 0.01 ^b^
	3	13.19 ± 0.23 ^c^	2.46 ± 0.09 ^c^	0.82 ± 0.05 ^c^
	RV	5.27–13.19	1.32–2.46	0.21–0.82
Clemenrubí	1	6.22 ± 0.02 ^b^	2.93 ± 0.01 ^b^	1.72 ± 0.01 ^c^
	2	5.73 ± 0.38 ^a^	3.98 ± 0.24 ^c^	0.40 ± 0.02 ^a^
	3	13.21 ± 0.42 ^c^	2.50 ± 0.05 ^a^	1.59 ± 0.07 ^b^
	Range of Variation	5.73–13.21	2.50–3.98	0.40–1.72
Clemensoon	1	7.12 ± 0.07 ^b^	3.61 ± 0.05 ^c^	0.21 ± 0.01 ^a^
	2	3.36 ± 0.14 ^a^	1.52 ± 0.06 ^a^	0.36 ± 0.08 ^b^
	3	10.98 ± 0.55 ^c^	2.43 ± 0.01 ^b^	2.24 ± 0.05 ^c^
	RV	3.36–10.98	1.52–3.61	0.21–2.24

RV: Range of variation. Mean ± Standard deviation (*n* = 9). In each column, the different letters (a,b,c) indicate statistically significant differences (*p* < 0.05) between seasons for each variety.

## Supplementary Materials

The following are available online at https://www.mdpi.com/2304-8158/9/5/642/s1, Table S1: Tentative identification of phenolic compounds in extra early clementine varieties, *Citrus clementina* Hort ex Tan. Table S2: Full quantification (mg/g of extract) of phenolic compounds in extra early clementine varieties, *Citrus clementina* Hort ex Tan. (mean ± SD, *n* = 9).
