# Supplementary material for: Characterization of Extra Early Spanish Clementine Varieties (Citrus clementina Hort ex Tan) as a Relevant Source of Bioactive Compounds with Antioxidant Activity
Source: Foods. 2020 May 16;9(5):642. doi: 10.3390/foods9050642 (PMC7278874; doi:10.3390/foods9050642)
Supplement: Supplementary file 1 [file foods-09-00642-s001.zip › foods-791927-SI.pdf]

Supplemental material

Table S1. Tentative identification of phenolic compounds in extra early clementine varieties, *Citrus clementina* Hort ex Tan.

Peak	Rt	λmax	Molecular ion [M-H]- (m/z)	MS2 (m/z)	Tentative identification
1	5.2	272	385	281(78), 223(5),193(12),133(10)	Sinapoyl-glucoside1
2	6.4	324	385	367(9),209(27),191(100),173(3),147(15),129(19)	Feruloyl-glucaric acid2
3	6.8	266	341	179(100),135(52)	Caffeoyl-hexoside3
4	9.1	274,sh328	355	295(2),235(4),217(13),193(28),175(100)	Feruloyl-6′-O-glucoside2
5	9.5	332	593	575(2),503(9),473(20),383(18),353(23)	Vicenin II (apigenin-6,8-di-C-glucoside)4
6	10.7	282,sh336	433	271(100)	Naringenin-O-hexoside5
7	10.9	284,sh338	443	399(3),381(4),341(2),299(14),281(15)	Dihydrophaseic acid glucoside
8	11.6	268	281	237(95),189(29),171(100),145(12),139(19),123(54)	Dihydrophaseic acid
9	13.0	338	623	605(3),533(5),503(31);413(11);383(22),312(8)	Diosmetin-6,8-di-C-glucoside6
10	14.9	286,sh336	427	385(7),367(27),223(9),205(50),179(10),153(100),138(19)	Hydroxycinnamoyl derivative7
11	16.9	326	561	367(100),337(10),193(84),191(33),173(29),134(18)	Dihydroferulic-feruloylquinic acid dimer2
12	17.4	326	561	367(100),337(8),193(92),191(29),173(26),134(14)	Dihydroferulic-feruloylquinic acid dimer2
13	18.2	356	609	301(100)	Rutin (quercetin-3-O-rutinoside)8
14	19.8	280,sh334	579	271(100)	Narirutin/naringin5
15	22.5	284,sh340	609	301(100)	Hesperidin/Neohesperidin9
16	23.1	348	693	633(2),595(9),507(3),395(4)	Nomilin-glucoside9
17	30.8	345	593	285(100)	Kaempferol-O-deoxyhexosyl-hexoside10

Retention time (Rt), wavelengths of maximum absorption in the visible region (λmax), mass spectral data.

Table S2. Full quantification (mg/g of extract) of phenolic compounds in extra early clementine varieties, *Citrus clementina* Hort ex Tan. (mean ± SD, n=9).

Season	1			2			3		
**Tentative identification**	**Basol**	**Clemenrubí**	**Clemensoon**	**Basol**	**Clemenrubí**	**Clemensoon**	**Basol**	**Clemenrubí**	**Clemensoon**
Sinapoyl-glucoside[1]	3.4 ± 0.2	2.04 ± 0.01	3.92 ± 0.01	1.09 ± 0.01	1.40 ± 0.04	0.88 ± 0.01	1.20 ± 0.02	0.93 ± 0.01	0.90 ± 0.01
Feruloyl-glucaric acid[2]	0.99 ± 0.01	1.01 ± 0.03	2.95 ± 0.05	0.49 ± 0.01	0.46 ± 0.03	0.58 ± 0.01	0.65 ± 0.03	0.62 ± 0.01	0.45 ± 0.02
Caffeoyl-hexoside[3]	1.56 ± 0.03	1.58 ± 0.07	3.26 ± 0.07	1.03 ± 0.02	0.90 ± 0.01	0.72 ± 0.01	1.09 ± 0.02	0.93 ± 0.03	0.85 ± 0.01
Feruloyl-6′-O-glucoside[2]	1.07 ± 0.01	0.68 ± 0.03	1.32 ± 0.05	0.15 ± 0.01	0.20 ± 0.01	0.19 ± 0.01	0.25 ± 0.01	0.23 ± 0.01	0.16 ± 0.01
Vicenin II (apigenin-6,8-di-C-glucoside)[4]	2.80 ± 0.01	0.88 ± 0.01	1.84 ± 0.01	0.42 ± 0.01	0.46 ± 0.01	0.37 ± 0.01	0.84 ± 0.01	0.69 ± 0.02	0.63 ± 0.01
Naringenin-O-hexoside[5]	0.80 ± 0.01	0.47 ± 0.02	1.38 ± 0.01	0.16 ± 0.01	0.17 ± 0.01	0.13 ± 0.01	0.18 ± 0.01	0.12 ± 0.01	0.09 ± 0.01
Dihydrophaseic acid glucoside	nq	nq	nq	nq	nq	nq	nq	nq	nq
Dihydrophaseic acid	nq	nq	nq	nq	nq	nq	nq	nq	nq
Diosmetin-6,8-di-C-glucoside[6]	0.54 ± 0.01	0.251 ± 0.005	0.50 ± 0.01	0.17 ± 0.01	0.22 ± 0.01	0.16 ± 0.01	0.20 ± 0.01	0.18 ± 0.01	0.12 ± 0.01
Hydroxycinnamoyl derivative[7]	0.14 ± 0.01	0.099 ± 0.001	0.217 ± 0.001	0.09 ± 0.01	0.09 ± 0.01	0.04 ± 0.01	0.11 ± 0.01	0.09 ± 0.01	0.05 ± 0.01
Dihydroferulic-feruloylquinic acid dimer[2]	1.32 ± 0.03	0.91 ± 0.01	1.671 ± 0.001	0.25 ± 0.01	0.20 ± 0.02	0.13 ± 0.01	0.14 ± 0.01	0.16 ± 0.01	0.09 ± 0.01
Dihydroferulic-feruloylquinic acid dimer[2]	2.23 ± 0.02	1.223 ± 0.003	2.597 ± 0.004	0.26 ± 0.02	0.27 ± 0.01	0.13 ± 0.01	0.24 ± 0.01	0.22 ± 0.01	0.16 ± 0.01
Rutin (quercetin-3-O-rutinoside)[8]	1.35 ± 0.01	0.48 ± 0.01	1.51 ± 0.01	0.23 ± 0.01	0.24 ± 0.01	0.32 ± 0.01	0.22 ± 0.01	0.36 ± 0.01	0.20 ± 0.01
Narirutin/naringin[5]	4.15 ± 0.01	2.563 ± 0.011	5.82 ± 0.01	0.80 ± 0.01	0.91 ± 0.01	0.75 ± 0.01	0.81 ± 0.01	0.76 ± 0.01	0.63 ± 0.01
Hesperidin/Neohesperidin[9]	4.52 ± 0.02	2.98 ± 0.02	5.25 ± 0.02	0.51 ± 0.01	0.65 ± 0.01	0.34 ± 0.01	0.59 ± 0.01	0.54 ± 0.01	0.39 ± 0.01
Nomilin-glucoside[9]	0.197 ± 0.001	0.056 ± 0.001	0.115 ± 0.001	0.04 ± 0.01	0.03 ± 0.01	0.04 ± 0.01	0.04 ± 0.01	0.04 ± 0.01	0.03 ± 0.01
Kaempferol-O-deoxyhexosyl-hexoside[10]	0.25 ± 0.01	0.12 ± 0.01	0.419 ± 0.001	0.25 ± 0.01	0.18 ± 0.01	0.14 ± 0.01	0.21 ± 0.01	0.18 ± 0.01	0.20 ± 0.01
**Total flavonoids**	13.81 ± 0.02b	7.33 ± 0.04c	15.46 ± 0.01a	3.36 ± 0.02b	3.53 ± 0.01a	2.66 ± 0.01c	3.67 ± 0.01a	3.17 ± 0.04b	2.66 ± 0.01c
**Total phenolic acids**	10.51 ± 0.50b	7.45 ± 0.21c	15.73 ± 0.07a	2.58 ± 0.02b	2.84 ± 0.02a	2.24 ± 0.01c	3.10 ± 0.01a	2.86 ± 0.05b	2.28 ± 0.02c
**Total phenolic compounds**	25.27 ± 0.53b	15.35 ± 0.21c	32.78 ± 0.08a	5.94 ± 0.01b	6.37 ± 0.01a	4.91 ± 0.01c	6.78 ± 0.01a	6.03 ± 0.09b	4.94 ± 0.03c

nq - not quantified. Calibration curves used: 1- sinapic acid (y=270.42x+62.29; R^2=0.999); 2- ferulic acid (y=525.36x+233.82; R^2=0.999); 3- caffeic acid (y=359.01x+488.40; R^2=0.998); 4- apigenin-6-*C*-glucoside (y=179.52x+116.83; R^2=0.999); 5- naringenin (y=539.98x+161.46; R^2=0.995); 6- luteolin-6-*C*-glucoside (y=423.95x+91.13; R^2=0.999); 7- cinnamic acid (y=1979x+787.15; R^2=0.994); 8- quercetin-3-*O*-rutinoside (y=280.87x+373.73; R^2=0.998); 9-hesperetin (y=792.22x-76.88; R^2=0.999); 10- kaempferol-3-*O*-rutinoside (y=182.94x+96.64; R^2=0.999). In each row per season different letters mean significant differences (p<0.05).
